# Serratus Anterior Plane Block for Procedural Anesthesia for Pigtail Tube Thoracostomy: A Case Series

**DOI:** 10.5811/cpcem.21251

**Published:** 2025-01-19

**Authors:** Edward Lopez, Raghav Sahni, Maxwell Cooper, Michael Shalaby

**Affiliations:** * Mount Sinai Medical Center, Department of Emergency Medicine, Miami Beach, Florida; † Drexel University College of Medicine, Department of Emergency Medicine, Philadelphia, Pennsylvania; ‡ Drexel University College of Medicine, Department of Emergency Ultrasound, Philadelphia, Pennsylvania; § Herbert Wertheim College of Medicine at Florida International University, Department of Emergency Medicine and Critical Care, Miami Beach, Florida

**Keywords:** chest tube, tube thoracostomy, serratus anterior plane block, ultrasound, regional anesthesia

## Abstract

**Introduction:**

Pneumothoraces are frequently treated by emergency physicians. Tube thoracostomy, the definitive treatment for a spontaneous pneumothorax, is associated with significant pain. Analgesia prior to tube thoracostomy often involves the administration of opioids and local infiltration of anesthetics. Thus far, regional anesthesia prior to pigtail tube thoracostomy in the emergency department (ED) has not been well described; it offers promise in alleviating pain associated with this procedure. Due to its ability to anesthetize all or most of the structures associated with tube thoracostomy—skin, serratus anterior muscles, intercostal muscles, and the parietal pleura—the serratus anterior plane block (SAPB) is a potentially promising fascial plane block prior to pigtail tube thoracostomy.

**Case Series:**

We present three cases of patients in the ED who received a SAPB and had nearly complete or complete anesthesia during pigtail tube thoracostomy.

**Conclusion:**

Pigtail tube thoracostomies are commonly performed in the ED and can be associated with significant pain despite a multimodal approach to pain management. The SAPB offers a safe and effective approach to anesthesia for patients in the ED undergoing a pigtail tube thoracostomy.

## INTRODUCTION

The pleural space is bordered by the parietal and visceral pleura, which are connective tissues that line the chest wall and the lungs, respectively. Small amounts of physiological fluid within the pleural space aids in lung sliding and expansion. Pneumothorax (PTX), a condition in which air infiltrates the pleural space and causes compression and deflation of the lung, is frequently encountered in the emergency department (ED).^1^–^5^ Pneumothoraces manifest in various forms, generally categorized as spontaneous or non-spontaneous, with primary (no known lung pathology) and secondary (underlying lung pathology) subtypes for spontaneous occurrences, and iatrogenic and traumatic origins for non-spontaneous cases.^4^ Changes in ambient atmospheric pressure, smoking, and connective tissue disorders, such as Marfan syndrome, are all potential factors that contribute to the creation of a PTX.^4^,^6^,^7^ Regardless of the etiology, tension physiology occurs when sufficient air enters the pleural cavity to cause hemodynamic compromise.^4^,^8^ While tension PTX requires immediate intervention to prevent life-threatening decompensation, smaller PTXs may allow emergency physicians time to take a more calculated approach, leading to greater patient comfort.^4^

Tube thoracostomy is a procedure in which a tube is placed (normally between the fourth and fifth intercostal spaces) to allow evacuation of air from the pleural space.^1^,^2^ En route to the pleural space, a chest tube enters through the skin and traverses the subcutaneous tissue, serratus anterior and intercostal muscles, and finally the parietal pleura. These structures have sensory innervation arising from the intercostal nerve and its lateral cutaneous branches, the long thoracic nerve, and the intercostal nerves.^2^,^9^–^11^ Despite its life-saving potential, tube thoracostomy often induces significant pain to structures innervated by these nerves, necessitating effective pain management strategies.^2^,^9^

Currently, emergency physicians often use a combination of local anesthesia and intravenous analgesics such as opioids.^9^ While thoracic wall regional anesthesia techniques such as the serratus anterior plane block (SAPB) have been extensively used for chest wall analgesia and anesthesia for thoracic surgeries, there is a lack of literature on its use in the ED for pigtail tube thoracostomy.^12^,^13^ The SAPB could play a key role in easing discomfort associated with pigtail tube thoracostomy.^9^ It is performed by placing a patient in the supine position with the ipsilateral arm extended with the chest wall exposed.

Using a high-frequency linear probe, the physician visualizes the lateral aspect of the fourth or fifth rib and directs a needle in-plane either between the latissimus dorsi and serratus anterior muscles (superficial technique), or between the serratus anterior muscles and the rib itself (deep technique), and then deposits local anesthetic (Image).^9^,^14^ Subsequently, anesthetic spreads to the lateral cutaneous branches of the second through ninth thoracic intercostal nerves along with the intercostal, long thoracic, and thoracodorsal nerves, depending on the volume of anesthetic applied and the area of administration.^8^,^9^,^14^,^15^ We present a series of three patients with spontaneous PTX for whom a SAPB provided significant analgesia or complete anesthesia prior to pigtail tube thoracostomy.

**CPC-EM Capsule**

What do we already know about this clinical entity?

*The serratus anterior plane block (SAPB) is a well-defined technique in regional anesthesia that has demonstrated effectiveness in anesthesia of the hemithorax*.

What makes this presentation of disease reportable?

*We present a technique for pre-procedural anesthesia for a common procedure performed in the emergency department*.

What is the major learning point?

*The SAPB is an effective technique that emergency physicians can use to reduce pain when performing pigtail tube thoracostomy*.

How might this improve emergency medicine practice?

*The SAPB can improve pain control and reduce the need for opioids in the emergency department*.

## CASE SERIES

### Case One

A 32-year-old male without significant past medical history presented to the ED with a chief complaint of chest pain and mild dyspnea. The patient denied any preceding trauma, air travel, or ocean dives. His vital signs were within normal limits and body mass index (BMI) was 19.2 kilograms per meter squared (kg/m^2^) (normal range: 18.5–24.9 g/m^2^). A chest radiograph (CXR) revealed a left-sided PTX affecting 20% of the lung. The patient consented to a SAPB, which was performed with 20 milliliters (mL) of bupivacaine 0.5% without epinephrine. The physicians instilled 10 mL of bupivacaine in the superficial plane (between the latissimus dorsi and the serratus anterior muscles) and 10 mL in the deep plane (between the serratus anterior muscles and the rib). The patient was then reassessed after roughly five minutes to assess for anesthesia. He was noted to have reached adequate anesthesia, after which insertion of the pigtail thoracostomy was initiated. Thereafter, the patient experienced no pain with the procedure, which was performed in the fourth intercostal space. A repeat CXR revealed appropriate placement of the pigtail tube. The patient was admitted and discharged the next day without any complications.

### Case Two

A 45-year-old male presented to the ED with chest pain and dyspnea. Other than daily cigarette smoking, the patient denied any past medical history, preceding trauma, air travel, or ocean dives. The patient’s heart rate was elevated to the low 100s beats per minute, but otherwise his oxygen saturation, respiratory rate, and blood pressure were within normal limits. His BMI was 28.9 kg/m^2^. A CXR demonstrated a right-sided PTX affecting 25% of the lung. The patient consented to a SAPB, which was performed with 20 mL bupivacaine 0.5% without epinephrine; 10 mL were instilled in the superficial plane and 10 mL in the deep plane. The patient was then reassessed after roughly five minutes to assess for anesthesia. The patient was noted to have reached adequate anesthesia, after which insertion of the pigtail thoracostomy was initiated. The patient complained of no pain during tube thoracostomy up to the intercostal muscles, but when the pigtail traversed the parietal pleura he complained of 2/10 pain. A repeat CXR revealed appropriate placement of the pigtail tube. The patient was admitted to the hospital and discharged the next day without any complications.

### Case Three

A 17-year-old male without significant prior medical history presented to the ED with a chief complaint of chest pain that began the preceding night. The patient endorsed left-sided chest pain that was aggravated by inspiration. On physical exam the patient had stable vital signs and was breathing comfortably with a 95% oxygen saturation on room air; but lung auscultation revealed decreased breath sounds on the left. The patient’s BMI was 19.8 kg/m^2^. The patient rated his pain as a 4/10, and 15 milligrams of intravenous ketorolac was administered. A subsequent CXR revealed a PTX. The patient and mother then consented to a left-sided SAPB prior to placement of a pigtail tube thoracostomy. A deep SAPB was performed with 15 mL of bupivacaine 0.5% with epinephrine. The patient was then reassessed after roughly five minutes to assess for anesthesia. The patient was noted to have reached adequate anesthesia, after which insertion of the pigtail thoracostomy was initiated. During the tube thoracostomy the patient denied any pain or discomfort. A repeat CXR revealed good tube placement after which the patient was admitted and then discharged on hospital day three without complications.

## DISCUSSION

Pneumothoraces are frequently treated by emergency physicians. Tube thoracostomy, a definitive treatment for a spontaneous PTX, is associated with significant pain.^1^,^2^,^4^,^5^ Analgesia prior to pigtail tube thoracostomy often involves the administration of systemic opioids and local infiltration of anesthetics.^9^ However, the rise of the opioid epidemic in the Western world has also bolstered the use of regional anesthesia in the ED. Thus far, regional anesthesia prior to pigtail tube thoracostomy in the ED has not been well described; it offers promise in alleviating physical suffering associated with this painful procedure.

Due to its ability to anesthetize all or most of the structures associated with pigtail tube thoracostomy—skin, serratus anterior muscles, intercostal muscles, and the parietal pleura—the SAPB is a potentially promising fascial plane block prior to pigtail tube thoracostomy.^9^,^14^,^15^ Furthermore, its shallow depth (even in patients with elevated BMI, as in Case Two) and straightforward technique make the SAPB accessible to emergency physicians familiar with needle-guided procedures and can be performed on both adults and pediatric patients alike, as demonstrated in this case series. Employing short-acting local anesthetics like lidocaine reduces the risk of local anesthetic systemic toxicity compared to bupivacaine, ensuring further safety during SAPB administration.^16^ Importantly, in cases where there is a pre-existing PTX, the risk of inadvertently advancing the needle into the pleura, a typical concern with SAPB, is no longer a concern, emphasizing a critical safety advantage of this technique.

The difference between analgesia and anesthesia with a SAPB prior to pigtail tube thoracostomy lies in its ability to anesthetize the parietal pleura, which is innervated by the intercostal nerve. The SAPB targets the lateral cutaneous branches of the intercostal nerves (Figure). It could be presumed that local anesthetic from a SAPB may not reach the intercostal nerve itself, which lies between the innermost and the internal intercostal muscles that traverse the space between ribs deep to the serratus muscles. However, clinically the SAPB may in fact target the intercostal nerve, as is seen in this case series. Herein lies the difference in the anesthesia obtained via SAPB and traditional methods (skin wheel and infiltration of the tube pathway), as anesthetizing these nerves allows for both procedural and post-procedural surgical level anesthesia—taking less than five minutes in our cases.

Additionally, SAPB carries the benefit of providing anesthesia for other pain-causing pathology that may be present, such as rib fractures. Individual patient characteristics such as neural and muscular anatomy or body habitus may determine the difference between complete anesthesia and significant analgesia with a SAPB. For example, in certain patients local anesthetic may diffuse past the serratus anterior muscle to reach between the internal and innermost intercostal muscles; or perhaps in some patients the parietal pleura also receives innervation from the lateral cutaneous branches of the intercostal nerve. All three patients had normal or only slightly elevated BMI; therefore, the patient in Case Two may have had intrinsic anatomical characteristics that prevented him from experiencing complete anesthesia due to the intercostal nerve not being anesthetized by the SAPB. Given the limited size of this case series, the true extent of involvement of the intercostal nerve and, thus, anesthesia of the parietal pleura is yet to be elucidated. However, even mild discomfort with pigtail tube thoracostomy with a low-risk, easily performed regional anesthesia technique marks a significant advancement in emergency medicine.

Additionally, use of the SAPB may allow emergency physicians to avoid the use of opioids, which are associated with nausea, vomiting, respiratory depression, and addiction.^9^ As with any fascial plane block that involves large amounts of local anesthetic, emergency physicians should safeguard patients against local anesthetic systemic toxicity, which consists of systemic uptake of local anesthetic and is toxic to the central nervous and cardiovascular systems and can result in a cascade of neurological and cardiovascular symptoms, the extremes of which include seizures and cardiac arrest.^16^ Emergency physicians should always employ ideal body-weight dosing, such as described in *Miller’s Basics of Anesthesia*, and keep patients on cardiopulmonary monitoring since the first signs of systemic uptake include tachyarrhythmias.^17^ Lastly, while two patients received both deep and superficial SAPB, studies have demonstrated no difference in pain levels with one or the other.^15^ Our third patient only received a deep SAPB and experienced complete anesthesia.

Here, we illustrate how emergency physicians trained in ultrasound-guided procedures performed SAPBs, achieving complete or near-complete anesthesia in three patients undergoing pigtail tube thoracostomy for PTX. Importantly, none of the patients experienced adverse events during or after the procedure. Given the practicality and safety of SAPB, we advocate for its wider adoption among emergency physicians for patients requiring tube thoracostomy due to stable, symptomatic, spontaneous pneumothoraces.

## CONCLUSION

Pigtail tube thoracostomies are commonly performed in the ED and can be associated with significant pain despite a multimodal approach to pain management. The serratus anterior pain block offers a safe and effective approach to anesthesia for patients in the ED undergoing a pigtail tube thoracostomy.

## Figures and Tables

**Figure:**
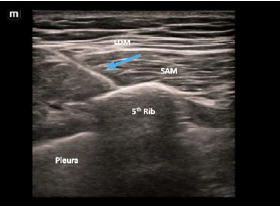
The structures involved in a serratus anterior plane block, including ribs with intercostal muscles, serratus anterior muscle lateral, and the latissimus dorsi muscle most superficial.

**Image:**
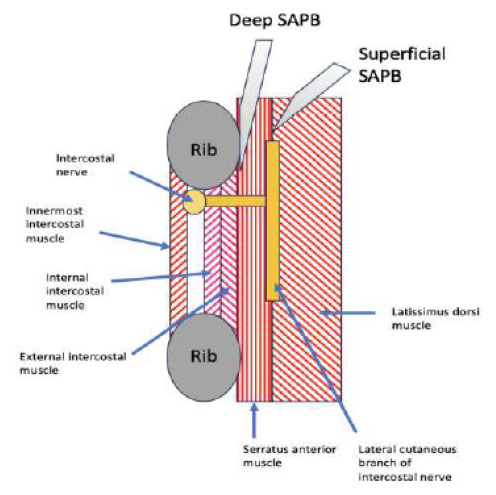
Ultrasound image of a serratus anterior plane block. The blue arrow denotes the needle shaft. *LDM*, latissimus dorsi muscle; *SAM*, serratus anterior muscle.

## References

[1] Dalbec DL, Krome RL (1986). Thoracostomy. Emerg Med Clin North Am.

[2] Kwiatt M, Tarbox A, Seamon MJ (2014). Thoracostomy tubes: a comprehensive review of complications and related topics. Int J Crit Illn Inj Sci.

[3] Charalampidis C, Youroukou A, Lazaridis G (2015). Pleura space anatomy. J Thorac Dis.

[4] Baumann MH, Noppen M (2004). Pneumothorax. Respirology.

[5] Roberts DJ, Leigh-Smith S, Faris PD (2015). Clinical presentation of patients with tension pneumothorax: a systematic review. Ann Surg.

[6] Scott GC, Berger R, McKean HE (1989). The role of atmospheric pressure variation in the development of spontaneous pneumothoraces. Am Rev Respir Dis.

[7] Hall JR, Pyeritz RE, Dudgeon DL (1984). Pneumothorax in the Marfan syndrome: prevalence and therapy. Ann Thorac Surg.

[8] Houston MC (1990). Pathophysiology of shock. Crit Care Nurs Clin North Am.

[9] Marshall K, McLaughlin K (2020). Pain management in thoracic surgery. Thorac Surg Clin.

[10] Patel SJ, Augoustides JGT (2020). Serratus anterior plane block: a promising technique for regional anesthesia in minimally invasive cardiac surgery. J Cardiothorac Vasc Anesth.

[11] Davies F, Gladstone RJ, Stibbe EP (1932). The anatomy of the intercostal nerves. J Anat.

[12] Lin J, Hoffman T, Badashova K (2020). Serratus anterior plane block in the emergency department: a case series. Clin Pract Cases Emerg Med.

[13] Khalil PA, Becker E (2022). Point-of-care ultrasound-guided serratus anterior plane block for chest tube placement in a spontaneous pneumothorax. Pediatr Emerg Care.

[14] Chen JQ, Yang XL, Gu H (2021). The role of serratus anterior plane block during in video-assisted thoracoscopic surgery. Pain Ther.

[15] Amaral S, Medeiros H, Lombardi R (2023). OP047 Going deep or staying superficial – which serratus anterior plane block wins for analgesia: a meta-analysis. Reg Anesth Pain Med.

[16] Dillane D, Finucane BT (2010). Local anesthetic systemic toxicity. Can J Anaesth.

[17] Berde C, Koka A, Drasner K, Pardo M (2018). Local anesthetics. Miller’s Basics of Anesthesia.

